# One-dimensional chain structures of hexanuclear uranium(iv) clusters bridged by formate ligands†

**DOI:** 10.1039/c8ra06330c

**Published:** 2018-10-12

**Authors:** Jie Ling, Huangjie Lu, Yaxing Wang, Kenndra Johnson, Shuao Wang

**Affiliations:** Department of Chemistry and Biochemistry, Claflin University Orangeburg SC 29115 USA jling@claflin.edu; State Key Laboratory of Radiation Medicine and Protection, School for Radiological and Interdisciplinary Sciences, Collaborative Innovation Center of Radiation Medicine of Jiangsu Higher Education Institution, Soochow University Suzhou 215123 China

## Abstract

Three one-dimensional chain structures of uranium(iv) hexanuclear clusters have been synthesized under hydrothermal/solvothermal conditions. Crystallographic studies disclose that the structures of [U_6_O_4_(OH)_4_(HCOO)_12_(H_2_O)]·3H_2_O (1a), [U_6_O_4_(OH)_4_(HCOO)_12_(HCOOH)(H_2_O)]·3H_2_O (1b) and (H_6_C_5_N)_2_[U_6_O_4_(OH)_4_(HCOO)_14_(H_5_C_5_N)] (2) contain a U(iv) hexanulear core [U_6_(*μ*_3_-OH)_4_(*μ*_3_-O)_4_]^12+^ which is decorated by terminal HCOO^−^ ligands and water (1a, 1b), HCOOH (1b) or pyridine molecules (2). These hexanuclear U(iv) clusters are further linked into zig–zag 1-D chain structures *via* bridging HCOO^−^ ligands. UV-vis-NIR spectra, together with bond valence calculations, indicate that all U atoms in three compounds exist as U(iv). Magnetic susceptibility data reveal that compound 2 exhibits paramagnetic characteristics.

## Abstract 2

Three one-dimensional chain structures of uranium(iv) hexanuclear clusters have been synthesized under hydrothermal/solvothermal conditions.

## Graphical abstract

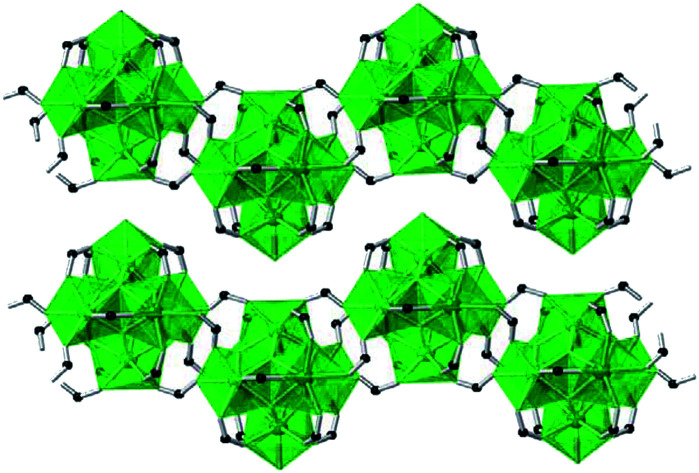

## Introduction

Actinide oxo clusters are of high interest because of their implication in nuclear waste disposal and actinide migration in the environment.^1–5^ For tetravalent actinides An(iv), it is known that they have strong tendencies to hydrolyze in aqueous solution and form a variety of actinide hydroxides An(OH)_*n*_^4−*n*^, which can further condense and result in the formation of polynuclear clusters, aggregates and even colloids.^6–8^ Such species may be highly mobile in aqueous system and dramatically influence the transport and migration of radionuclides in the environment as has been demonstrated for Pu(iv).^9–11^ U(iv), the major component in nuclear fuel, it can form soluble polynuclear clusters rather than insoluble UO_2_ by bioreduction of hexavalent U(vi) with bacterias.^12,13^

The first structurally characterized high nuclearity U(iv) cluster, U_6_(OH)_8_((C_6_H_5_O)_2_PO_2_)_12_, was reported by Mokry in 1996, and it was synthesized by reacting uranyl acetate with diphenylphosphate and [TpVCl_2_(dmf)] (Tp = hydridotris(pyrazolyl)borate) in acetonitrile under inert atmosphere.^14^ After that, several U(vi) clusters containing 6 to 38 uranium atoms were discovered in various organic solvents under inert atmosphere by reducing U(vi), oxidizing U(iii) or using U(iv) directly. Berthet synthesized four hexanuclear U(iv) clusters, [U_6_O_8_(OTf)_8_(Py)_8_], [U_6_O_8_I_8_(Py)_10_], [U_6_O_8_(OTf)_8_(THF)_8_], and [U_6_O_8_(*η*^8^-C_8_H_8_)(OTf)_6_(Py)_8_], by reacting [U(*η*^8^-C_8_H_8_)_2_] with [UO_2_(OTf)_2_] or [UO_2_I_2_(THF)_3_] in pyridine or THF solution.^15^ Nocton reported a hexanuclear U(iv) cluster [U_6_O_4_(OH)_4_(*η*-dbm)_12_] and a dodecanuclear U(iv)/U(v) cluster [U_12_(OH)_8_(O)_12_I_2_(OTf)_16_(CH_3_CN)_8_] which were synthesized by reacting UI_3_(OTf)_4_ with dbm or triflate in acetonitrile.^16^ Biswas synthesized two large U(iv) clusters, decanuclear [U_10_O_8_(OH)_6_(bz)_14_I_4_(H_2_O)_2_(CH_3_CN)_2_] and hexadecanuclear [U_16_O_22_(OH)_2_(bz)_24_], by reacting UI_3_(THF)_4_ with benzoic acid (bz) in acetonitrile solution.^17^ Falaise made the largest U(iv) cluster, [U_38_O_56_Cl_18_(THF)_8_(bz)_24_], from a solvothermal reaction of UCl_4_ and benzoic acid in THF solvent.^18^ These U(iv) clusters can be also synthesized in aqueous solution with the presence of N_2_H_4_ as a reducing agent. Takao reported the first formate hexanuclear U(iv) clusters, [U_6_O_4_(OH)_4_(HCOO)_12_(H_2_O)_6_] and [U_6_O_4_(OH)_4_(HCOO)_12_(H_2_O)_3_(CH_3_OH)_3_], by reacting U(ClO_4_)_4_ with HCOOH in acidic aqueous condition.^19^ Tamain discovered three An(iv) (An = U, Np, Pu) hexanuclear clusters and the U(iv) cluster, [U_6_(OH)_4_O_4_(H_2_O)_8_(HDOTA)_4_], was made by reacting U(NO_3_)_4_ with tetraazacyclododecane-1,3,7,10-tetraacetic acid (DOTA) in acidic solution.^20^ Diwu reported four An(iv) (An = Th, U, Np) hexanuclear clusters, including two U(iv) clusters, (NH_4_)_4_U_12_Cs_8_[C_6_H_4_(PO_3_)(PO_3_H)]_12_(NO_3_)_24_·18H_2_O and (NH_4_)_4_U_12_Cs_2_[C_6_H_4_(PO_3_)(PO_3_H)]_12_(NO_3_)_18_·40H_2_O made by reacting UCl_4_ and 1,2-phenylenediphosphonic acid at room temperature.^21^ Rather than typical octahedral clusters, those two diphosphonate hexanuclear U(iv) clusters adopt a planar six-membered ring structure. These discrete U(iv) clusters have also been extended into infinite two- and three-dimensional structures, for instance, [U_6_O_4_(OH)_4_(H_2_O)_6_(L)_6_] (L = 4,4′-bpdc, 2,6-ndc, 1,4-bdc, and fum),^22^ [U_6_O_8_(*μ*_2_-OTf)_8_(*η*_2_-OTf)_4_] and [U_6_O_8_(*μ*_2_-OTf)_12_(H_2_O)_3.5_],^23^*via* the linkage of carboxylate and trifluoromethanosulfonate ligands.

In aim to expand the family of U(iv) polynuclear clusters, we explored the hydrothermal reactions of uranyl acetate with formic acid under alkaline aqueous solution with the presence of reducing agent and discovered three one-dimensional chain structures of hexanuclear U(iv) core, [U_6_O_4_(OH)_4_(HCOO)_12_(H_2_O)]·3H_2_O (1a), [U_6_O_4_(OH)_4_(HCOO)_12_(HCOOH)(H_2_O)]·3H_2_O (1b) and (H_6_C_5_N)_2_[U_6_O_4_(OH)_4_(HCOO)_14_(H_5_C_5_N)] (2). To our best knowledge, these are the first one-dimensional structures of U(iv) clusters. Their syntheses, crystal structures, adsorption spectral and magnetic properties have been investigated and discussed in this work.

## Experimental

### Syntheses

**Caution**: While the UO_2_(OAc)_2_·2H_2_O used in these experiments contains isotopically depleted U, standard precautions for handling radioactive materials should be followed.

UO_2_(OAc)_2_·2H_2_O (MV Laboratories, Lot no. P705UA1), HCOOH (95%, Alfa-Aesar), Na_2_CO_3_ (99%, Sigma-Aldrich), and pyridine (99%, VWR) were used as received. Distilled and Millipore filtered water with a resistance of 18.2 MΩ cm was used. Zn amalgam was prepared in the laboratory by mixing an approximate ratio by weight of 30% Zn: 70% Hg in a round-bottom flask. The round-bottom flask was heated on an oil bath pan at 180 °C until the Zn completely dissolved into the Hg.^24^ Amalgams were recovered after reactions and reused in subsequent reactions until lost enough Zn to render them unusable.

In all reactions, Zn amalgam was added as a reducing agent for the reduction of U(vi) to U(iv). Compound 1a and 1b were synthesized as co-products from a hydrothermal reaction of UO_2_(OAc)_2_·2H_2_O (0.085 g, 0.2 mmol), Na_2_CO_3_ (0.064 g, 0.6 mmol), HCOOH (1 ml, 21.7 mmol), 1 ml H_2_O, and 7 g Zn amalgam. This reaction was sealed in a 23 ml PTFE-lined autoclave, heated at 150 °C in a box furnace for 2 hours, and slowly cooled down to room temperature in 12 hours. The products, green rhombohedra crystals, were washed with water and ethanol and left to dry in the air. The yields for compound 1a and 1b were not measured due to the difficulty of separating those two phases.

Compound 2 was synthesized under a similar condition as compound 1a and 1b except 1 ml pyridine was used as solvent instead of 1 ml water. Rod-like green crystals of compound 2 were obtained as single phase product with an approximate yield of 30% based on U.

### Crystallographic studies

Single crystals of 1a, 1b and 2 were selected using a polarized-light stereomicroscope and mounted on tapered glass fibers with epoxy for X-ray diffraction analysis. A sphere of diffraction data was collected for each compound at room temperature using a Bruker APEX DUO diffractometer equipped with an APEX CCD detector. The data were collected using monochromated Mo Kα X-ray radiation with a frame width 0.5° in *ω* and an exposure time per frame 20 second. Unit cell parameters were refined by least-squares techniques using the Bruker SMART software.^25^ The SAINT software^26^ was used for data integration including Lorentz, background and polarization corrections. Empirical absorption corrections were applied using the SADABS program.^27^ SHELXTL was used for the solution and refinement of the crystal structures.^28^ Atomic coordinates and additional structural information are provided in the cif files which can be accessed for free at the Cambridge Crystallographic Data Center (CCDC) with deposition numbers 1850747 (compound 1a), 1850748 (compound 1b) and 1850749 (compound 2). Selected data collection parameters and crystallographic information are listed in Table 1.

**Table 1:** Selected crystallographic data for compound 1a, 1b and 2

Compound	1a	1b	2
Cryst syst	Monoclinic	Triclinic	Monoclinic
Space group	*C*2/*c*	*P*1̄	*C*2/*c*
*a* (Å)	20.236(3)	11.5396(5)	20.458(3)
*b* (Å)	11.821(2)	11.7744(8)	15.473(3)
*c* (Å)	16.322(2)	16.2574(8)	15.918(2)
*α* (deg)		77.994(3)	
*β* (deg)	104.698(4)	77.951(2)	107.718(10)
*γ* (deg)		60.742(2)	
*V* (Å^3^)	3776.7(9)	1870.1(2)	4799.9(13)
*Z*	4	2	4
*D*_calcd_	3.895	3.893	3.360
*T* (K)	296	296	296
*λ* (Å)	0.71073	0.71073	0.71073
*F*(000)	3816	1868	4272
*θ*-max (deg)	27.52	30.157	27.44
Mu	25.760	26.008	20.283
Reflns	4221	10 777	5569
Indep. reflns	3292	7370	4179
*R*_1_ (%)	3.51	4.38	2.96
w*R*_2_ (%)	6.52	9.01	5.85
*S*	1.07	0.90	0.95

### UV-vis-NIR absorption spectroscopy

UV-vis-NIR absorption spectra for all three compounds were acquired from single crystals using a Craic Technology microspectrophotometer. Crystals were placed on a glass slide under Krytox oil and the data were collected from 300 nm to 1700 nm at ambient condition. The exposure time was auto-optimized by the instrument software, and spectra were background corrected for the slide and oil.

### Powder X-ray diffraction

Powder pattern of ground crystalline samples obtained from the reactions of compound 1a and 1b, and compound 2 were collected from 5° to 50°, with a step of 0.02° using a Bruker D8 advance X-ray diffractometer with Cu-Kα radiation (*λ* = 1.54056 Å) equipped with a Lynxeye one-dimensional detector.

### Magnetic property measurement

Magnetic property of compound 2 was analysed by a Quantum Design MPMS-XL SQUID magnetometer. Pure samples of ground crystals of compound 2, checked by PXRD, were weighted on a balance sensitive to 0.01 mg and loaded into a SQUID magnetometer. Its magnetic susceptibility data were measured in an applied field of 0.1 T in the temperature range of 2 to 300 K. The magnetic properties of compound 1a and 1b were not examined due to the failure of getting enough pure phases.

## Results and discussion

### Syntheses

Several attempts were conducted to get pure phases of compound 1a and 1b through adjusting mole ratio of reactants, pH of solution, reaction temperature and time, and amount of water, but no pure phase of either one could be obtained.

### Powder X-ray

Powder X-ray diffraction was used to check the composition of prepared sample. The experimental PXRD pattern of sample obtained from the reaction for compound 2 matches the simulated pattern calculated from the cif file of the structure with program CrystalMaker and it reveals that it is a single phase without any impurity (Fig. S1†).

### Crystallographic description

**Fig. 1:**
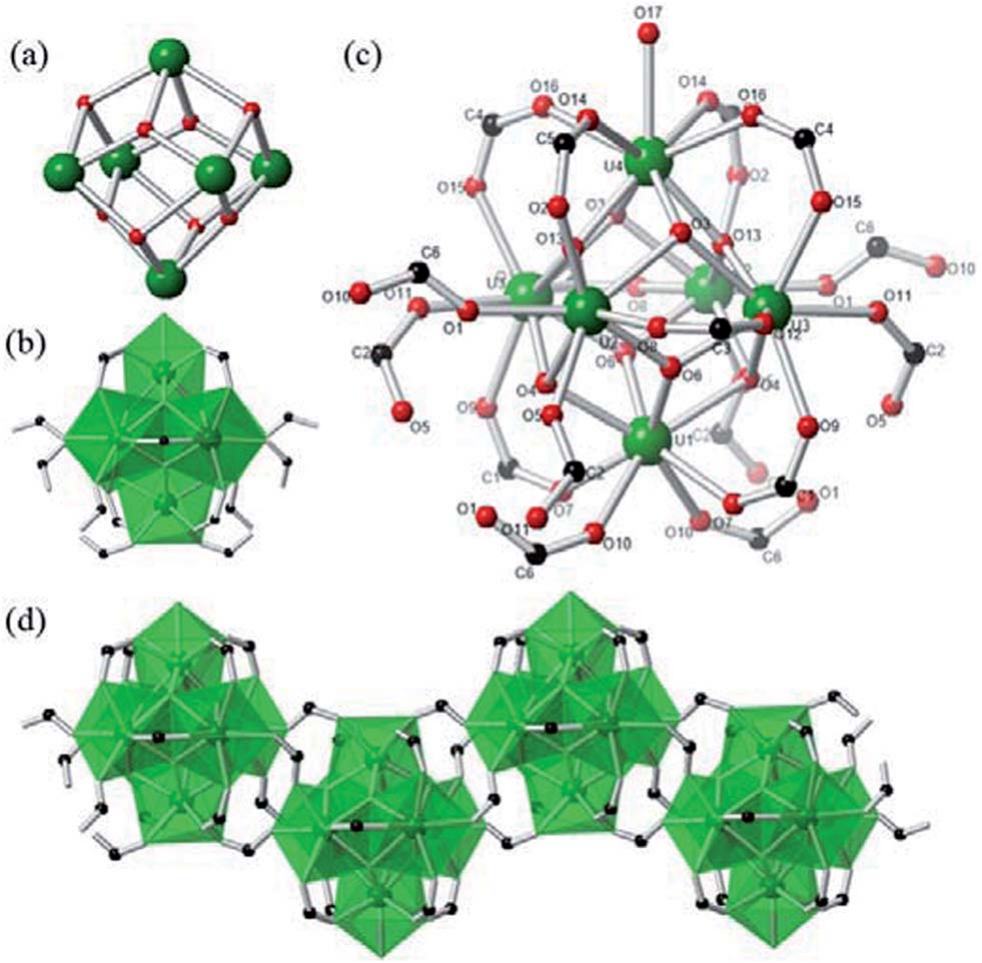
Crystal structure of compound 1a. (a) Hexanuclear U(iv) core achieved by *μ*_3_-O and *μ*_3_-OH; (b) polyhedra representation of core decorated by formate ligands; (c) ball-sticks representation of core structure; (d) 1D zig–zag chain of U(iv) clusters (color scheme: U, green; O, red; C, black).

Single crystal X-ray diffraction reveals that the structures of 1a, 1b, and 2 each contain a typically hexanuclear core as observed in previously reported hexanuclear U(iv) clusters.^14–20,22^ Within the hexanuclear core, six uranium atoms are arranged in a slightly distorted octahedral coordination geometry with U–U distances ranging from 3.790–3.875 Å and they are bridged by eight *μ*_3_-O sites which cap eight faces of the octahedron (Fig. 1a). As reported in previous hexanuclear U(iv) clusters, those eight *μ*_3_-O sites could be all O^2−^ groups, or all OH^−^ groups, or half O^2−^ and half OH^−^ groups.^14–17,19,20,22^ Although it is difficult to locate the hydrogen atoms of these O sites and differentiate OH^−^ from O^2−^ groups directly from electron density map due to the presence of heavy U atoms, those two groups can be distinguished by the U–O bond distance. According to literature, U(iv)–O distance are restricted to values between 2.21 and 2.27 Å, whereas the U(iv)–OH distances are substantially longer and have a range from 2.41 to 2.48 Å.^16,17,19,20,22^ In the structure of each compound, there are four O sites with U(iv)–O bond distances falling into the range of U(iv)–OH bond distance, where other four sites with U(iv)–O bond distances match that of U(iv)–O bond distances. The assignment of OH^−^ and O^2−^ groups are also confirmed by bond valence calculations carried out for these O atoms using Brown's parameter (Table S1†).^29^ The oxidation state of U(iv) ions in all three structure are confirmed by bond valence calculations using Burns' parameter.^30^ As a consequence, the hexanuclear core has a composition of [U_6_O_4_(OH)_4_]^12+^.

Compound 1a crystallizes in a monoclinic space group *C*2/*c* and contains four crystallographically unique uranium(iv) centers, four *μ*_3_-O/OH sites, six formate groups, and four water molecules. Four U(iv) centers have mixed coordination environments. Each U1, U2, and U3 atom is eight-coordinated by O atoms and results a UO_8_ polyhedron which has a standard *D*_4d_ square antiprism coordination geometry. Those U atoms are bounded to two *μ*_3_-oxo (2.187–2.244 Å), two *μ*_3_-hydroxo (2.419–2.468 Å) and four carboxylate O atoms from four HCOO^−^ groups (2.399–2.449 Å). Each U4 atom adopts a nine-coordinated UO_9_ capped square antiprism polyhedron in which U atom is bound to two *μ*_3_-oxo (2.297 Å), two *μ*_3_-hydroxo (2.427 Å), four formate O atoms (2.458–2.468 Å) and one terminal coordination water O atom (2.460 Å). Six unique HCOO^−^ groups adopt two coordination modes to U(iv) centres: C1, C3, C4, and C5 formates are bound to two U(iv) polyhedra within clusters in a syn–syn mode and serve as terminal ligands, while C2 and C6 formates adopt a syn–anti mode and they act as bridges to connect two U(iv) polyhedra from two adjacent hexanuclear clusters. Six U(iv) polyhedra are joined together by sharing the *μ*_3_-O/OH edges of one polyhedral with four neighbouring polyhedral and form a [U_6_O_4_(OH)_4_]^12+^ hexanuclear U(iv) core (Fig. 1a). These hexanuclear cores are further decorated by syn–syn formates (C1, C3, C4, C5) groups and linked together by syn–anti formates (C2, C6) (Fig. 1b and c), and as a consequence, result an infinitely zig–zag one-dimensional chain structure [U_6_O_4_(OH)_4_(HCOO)_12_(H_2_O)] (Fig. 1d). Three free waters are found in the open space between the chains and make an overall composition of [U_6_O_4_(OH)_4_(HCOO)_12_(H_2_O)]·3H_2_O for compound 1a.

**Fig. 2:**
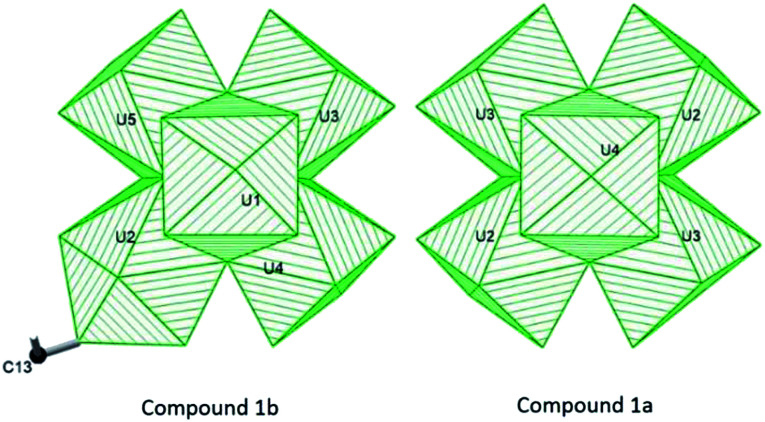
Coordination geometry of hexanuclear U(iv) cores in compound 1b (left) and 1a (right).

Compound 1b crystallizes in a triclinic *P*1̄ space group. In the structure of compound 1b, there are six unique uranium(iv) centers, eight *μ*_3_-O/OH sites, 13 formate groups, and four water molecules. U(iv) centers, U3, U4, U5, and U6, adopt the standard *D*_4d_ square antiprism coordination geometry and coordinate with eight oxygen atoms belonging to two *μ*_3_-oxo (2.187–2.242 Å), two *μ*_3_-hydroxo (2.408–2.468 Å) and four carboxylate O atoms from four HCOO^−^ groups (2.387–2.442 Å). U1 and U2 are both nine-coordinate and adopt a similar capped square antiprism geometry, but they display different coordination environments. U1 is surround by two *μ*_3_-oxo (2.297 Å), two *μ*_3_-hydroxo (2.427 Å), four formate O atoms (2.458–2.468 Å) and one terminal coordination water O atom (2.460 Å), while U2 doesn't have any coordinated terminal water, instead has a terminal monodentate formate (C13) ligand (Fig. 2). The long bond distances of U2–O35 (2.877(14) Å) and C–O (1.40(3) Å), as well as the bond valence calculations on the O34 and O35 atoms, indicate that C13 formate ligand is a neutral HCOOH molecule. Thirteen formate units are found in the structure and they adopt three different coordination modes to U(iv) centers; C1, C3, C4, C5, C7, C9, C10, C11 formates act as decorating bidentate ligands for hexanuclear core in a syn–syn mode, C2, C6, C8, C12 formates serve as bridging ligands for connecting adjacent hexanuclear cores, and C13 formate, as a formic acid molecule, attaches to U(2) as a terminal monodentate ligand. There are also three free water molecules found in the open space of chain structure and it results an overall composition of [U_6_O_4_(OH)_4_(HCOO)_12_(HCOOH)(H_2_O)]·3H_2_O.

**Fig. 3:**
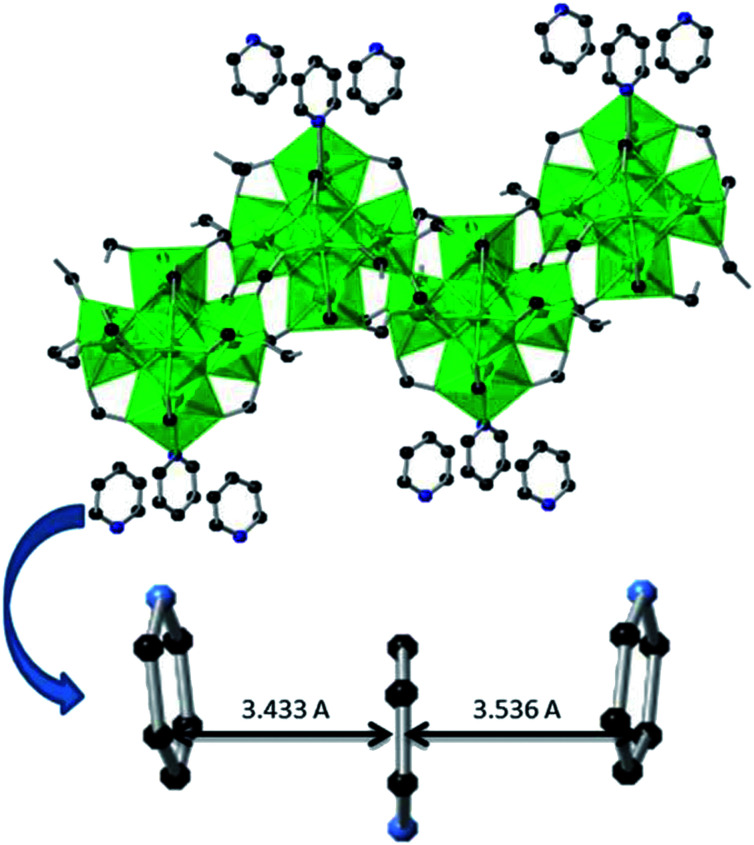
1D chain of U(iv) clusters and packing mode of pyridine ligands in compound 2 (U, green; O, red; C, black; N, blue).

Compound 2 crystallizes in a monoclinic space group *C*2/*c* and it contains four unique U(iv) centers, four *μ*_3_-O/OH sites, seven formate groups, and two pyridine groups. Among four U centers, three centers, U1, U2, and U3, adopt an eight-coordination square antiprism, while the other, U(4), has a nine-coordination capped square antiprism geometry. Within the UO_8_ polyhedra, U(iv) ion is coordinated by two μ_3_-oxo (2.219–2.255 Å), two μ_3_-hydroxo (2.390–2.464 Å) and four carboxylate O atoms from four HCOO^−^ groups (2.319–2.466 Å). For the nine-coordinated U4, the H_2_O site of UO_9_ polyhedron in compound 1a is occupied by one N atom of pyridine molecule with a U–N bond distance 2.769(8) Å. The remaining vertices of this capped square antiprism are occupied by two *μ*_3_-oxo (2.288 Å), two *μ*_3_-hydroxo (2.497 Å) and four carboxylate O atoms from four HCOO^−^ groups (2.415–2.425 Å). Seven formate units adopt three coordination modes to U(iv) centers. C3, C5, C8 formates act as decorating ligands for hexanuclear core in a syn–syn mode, C2 and C4 formates serve as bridging ligands for connecting adjacent hexanuclear cores, while C1 and C6 formates attach to U(iv) centers as terminal ligands in a monodentate coordination mode. There are two protonated pyridine groups found in the open space of chain structure and they are stacked together with the coordinated pyridine molecule in an antiparallel-displaced geometry owing to the π–π interaction^31–33^ with interplanar separation distances 3.433 to 3.536 Å (Fig. 3). This arrangement can be explained by the minimization of electric repulsion between two protonated N atoms in free pyridine ligands and the partially positively charged N atom in the coordinated pyridine ligand. Overall, compound 2 has a composition of (H_6_C_5_N)_2_[U_6_O_4_(OH)_4_(HCOO)_14_(H_5_C_5_N)].

### Structure comparison

Although three compounds adopt similar zig–zag one-dimensional chain structures of hexanucler U(iv) clusters, the U centers and formate groups display some distinct coordination environments.

By comparing the formulas of 1a, [U_6_O_4_(OH)_4_(HCOO)_12_(H_2_O)]·3H_2_O, and 1b, [U_6_O_4_(OH)_4_(HCOO)_12_(HCOOH)(H_2_O)]·3H_2_O, it is notable that compound 1b contains one extra HCOOH ligand. As depicted in Fig. 2, the extra HCOOH molecule in compound 1b, marked as C13 formate group, attaches to U2 and results a UO_9_ polyhedra rather than a UO_8_ polyhedra in compound 1a. Although the introduction of an extra formic acid molecule doesn't change the dimension of the chain structure too much, it decreases the symmetry of chain structure from a monoclinic *C*2/*c* space group in compound 1a to a triclinic *P*1̄ space group in compound 1b.

It was described in the experimental section that compound 2 was made under similar synthetic conditions as compounds 1a and 1b except using pyridine as a solvent instead of water. As a consequence, the water groups within compound 1a and 1b should be replaced by pyridine groups. As expected, single crystal XRD reveals that the water molecule sites within the hexanuclear cores in compound 1a and 1b are substituted by pyridine groups (Fig. 3). The free water molecules in compound 1a and 1b are also replaced by pyridine groups. The introduction of pyridine group increases the dimension of clusters. As seen in Table 1, compounds 1a and 2 crystallize in the same monoclinic *C*2/*c* space group, but compound 2 has a larger value of *b* (compound 1a: *b* = 11.812; compound 2: *b* = 15.473) and it is due to the alignment of pyridine groups along the *y* axis.

In all three chain structures, hexanuclear U(iv) clusters contain both eight- and nine-coordinated U(iv) polyhedra and those polyhedra are connected by edge-sharing *μ*_3_-O/OH groups. UO_8_ polyhedra from adjacent clusters are further linked together through syn–anti formate groups and result a chain structure aligning along the *z* direction. While for the nine-coordinated UO_9_ or UO_8_N polyhedron, the coordinated water (compound 1a and 1b) or pyridine (compound 2) molecule, acting as a terminal ligand, prevents the extension of clusters on the *xy* plane. This mixed coordination numbers of U(iv) within the same cluster are not observed in any previously reported U(iv) polynuclear clusters.^14–23^ For the clusters containing large organic ligands, such as diphenylphosphate,^14^ benzoate,^17,18^ DOTA,^20^ OTf,^15,23^ and dbm,^16^ the U(iv) ions usually adopt a UO_8_ polyhedra; While for small ligands, such as formate, U(iv) can have a UO_9_ polyhedra as found in the structure of [U_6_O_4_(OH)_4_(HCOO)_12_(L_t_)_6_] (L_t_ = H_2_O or CH_3_OH).^22^ UO_9_ polyhedra is also seen in the phenylenediphosphonate incorporated hexanuclear U(iv) clusters, (NH_4_)_4_U_12_Cs_8_[C_6_H_4_(PO_3_)(PO_3_H)]_12_(NO_3_)_24_·18H_2_O and (NH_4_)_4_U_12_Cs_2_[C_6_H_4_(PO_3_)(PO_3_H)]_12_(NO_3_)_18_·40H_2_O, which may be due to the lesser compact arrangement of U(iv) polyhedral as a planar six-membered ring.^21^ The coordination mode of ligands also plays a vital role on the structure of U(iv) clusters. All ligands in the previous reported U(iv) clusters adopt a syn–syn coordination mode to uranium metals and they serve as decorating ligands for the polynuclear core.^14–23^ While in the structures of three compounds reported here, formate ligands can have both syn–syn and syn–anti coordination modes and the later coordination mode enable the linkage of clusters and results the formation of extended chain structures of clusters.

### UV-vis-NIR absorption

U(iv), which has a [Rn]5f^2^ electron configuration, is known to produce a series of weak Laporte-forbidden f–f transitions in UV-vis-NIR region. In most cases, these transitions show relatively small variations with changing coordination environment, and they can be used as fingerprints to identify the oxidation states of uranium.^34,35^

**Fig. 4:**
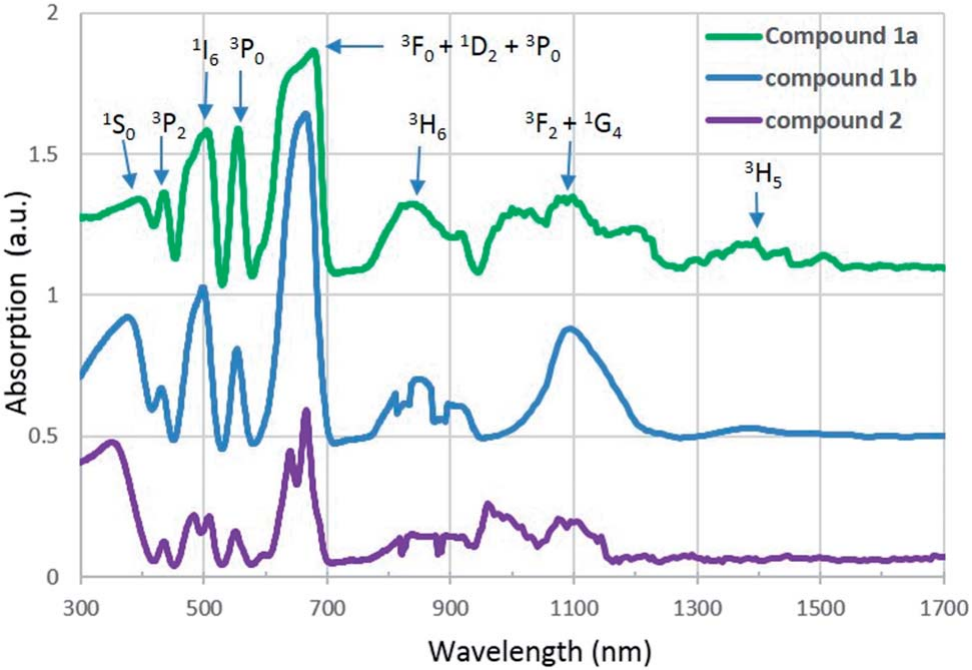
UV-vis-NIR absorption spectra of compound 1a, 1b and 2.

UV-vis-NIR absorption spectra of compound 1a, 1b and 2 are arranged from top to bottom in Fig. 4. For all three compounds, the strongest absorption bands are observed in the range of 580 to 709 nm and they can be assigned to transitions of the ^3^H_4_ ground state of U^4+^ to the mixture energy levels of ^3^F_4_, ^1^D_2_ and ^3^P_0_. The band in the lowest spectral region of 300 to 400 nm is attributed to transition from the ^3^H_4_ ground state to the ^1^S_0_ excited state. Three absorption bands located around 435, 504 and 556 nm correspond to the multiplets of ^3^P_2_, ^1^I_6_ and ^3^P_1_. In addition, two sets of absorption bands occuring in the regions 760–940 nm and 940–1260 nm are due to the transition of U^4+^ from ground state to the multiplet of ^3^H_6_ and a mixture of several levels of ^3^F_3_ and ^1^G_4_. In contrast to compound 2, an extra peak at 1400 nm is found in the spectra of compound 1a and 1b and it corresponds to the multiplet of ^3^H_5_. All of these absorption values are in agreement with those of U^4+^ in the literature.^21,36^

### Magnetic properties

Different from U(vi) (5f^0^), U(iv), a 5f^2^ system, typically exhibits a paramagnetic property arised from a combination of spin–orbital interactions and crystal field effects.^37,38^ Magnetic properties of many typical U(iv) compounds, including mono- or bi-nuclear and regular extended structures, have been well characterized,^38^ while that of polynuclear complexes are rarely investigated.^16–18,23^ In addition, the magnetic property, such as magnetic susceptibility data, can be used to identify the oxidation states of U in those compounds.

**Fig. 5:**
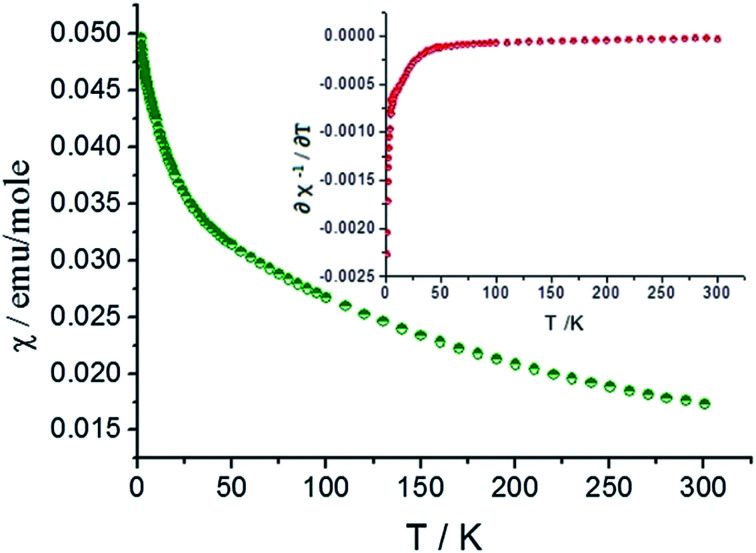
Magnetic susceptibility curve of compound 2.

The magnetic susceptibility data for compound 2 was collected over the temperature range of 2–300 K in an applied filed of 0.1 T. No differences were observed between the zero-field cooled (ZFC) and field cooled (FC) data. As shown in Fig. 5 (green curve), compound 2 shows paramagnetic characteristics and *χ* increases with decreasing the temperature.

In the high temperature region above 150 K (Fig. S2 and S3†), the magnetic susceptibility curve follows the Curie–Weiss(C–W) law. The inverse susceptibility data above 150 K was fitted to the C–W law (*χ* = *C*/(*T* − theta)), resulting in values of 8.790 emu K mol^−1^ and −248.07 K for the Curie and Weiss constants, respectively. From the Curie constant, an effective magnetic moment of *μ*_eff_ = 3.42 *μ*_b_ per U^4+^ ion at 300 K is obtained and it is slightly lower than 3.58 *μ*_b_ calculated from Russell–Saunders coupling of a ^3^H_4_ ground state. This is normally for U(iv) oxides^39,40^ and the value falls into the range of previously reported effective magnetic moment for other mononuclear and polynuclear U(iv) complexes (2.5–3.55 *μ*_b_)^41,42^.

Below 150 K, the magnetic susceptibility curve deviates from Curie–Weiss law and it is mainly due to the formation of f^2^ electronic singlet of U(iv) at low temperature and the population eliminating process of thermally excited state. As shown in Fig. S4,† the *χT* value approaches zero at low temperature and it is characteristic feature for U^4+^ ions.^38^ The differential results of the function relation between reversed magnetic susceptibility (*χ*^−1^) and temperature (inserted graph, red curve) indicate the transition from the f^2^ electronic triplet to singlet of U(iv) take place at 25 K.

## Conclusions

Three one-dimensional chain structures of uranium(iv) hexanuclear clusters have been synthesized under hydrothermal or solvothermal conditions by reacting U(vi) with formic acid in the presence of Zn amalgam. Single crystal XRD reveals that the six eight- or nine-coordinated U(iv) centers are joined together through *μ*_3_-O and *μ*_3_-OH groups and result a hexanuclear core. Those U(iv) hexanuclear cores are decorated by terminal formate ligands and further linked into one-dimensional chain structures by bridging formate ligands. UV-vis-NIR spectra of compounds 1a, 1b and 2 show characteristic U(iv) peaks. The magnetic susceptibility data of compound 2 indicate that it exhibits paramagnetic characteristics.

## Conflicts of interest

There are no conflicts to declare.

## Supplementary Material

RA-008-C8RA06330C-s001

RA-008-C8RA06330C-s002
