# Supplementary material for: One-dimensional chain structures of hexanuclear uranium(iv) clusters bridged by formate ligands
Source: RSC Adv. 2018 Oct 12;8(61):34947–53. doi: 10.1039/c8ra06330c (PMC9087207; doi:10.1039/c8ra06330c)
Supplement: RA-008-C8RA06330C-s001 [file RA-008-C8RA06330C-s001.pdf]

Electronic Supplementary Material (ESI) for RSC Advances.
This journal is © The Royal Society of Chemistry 2018

Supporting information for

One-Dimensional Chain Structures of Hexanuclear Uranium(IV) Clusters Bridged by Formate Ligands

Jie Ling,*[a] Huangjie Lu,[b] Yaxing Wang,[b] Kenndra Johnson,[a] Shuao Wang[b]

a: Department of Chemistry and Biochemistry, Claflin University, Orangeburg, SC, 29115, United States

b: State Key Laboratory of Radiation Medicine and Protection, School for Radiological and Interdisciplinary Sciences and Collaborative Innovation Center of Radiation Medicine of Jiangsu Higher Education Institution, Soochow University, Suzhou, 215123, China.

Table of Contents

S1. Powder X-ray diffraction of samples obtained from the reactions of compound 2 (Figure S1).

S2. Bond lengths between U(IV) and μ_3-O or μ_3-OH groups in three compounds (Table S1).

S3. Bond valence calculation data for μ_3-O and μ_3-OH groups in three compounds (Table S2).

S4. Magnetic susceptibility of compound 2. (1) χ^{-1} - T plot for compound 2 under the applied filed of 0.1 T (Figure S2). (2) The inverse susceptibility data above 150 K was fitted to the C–W law (Figure S3). (3) χT-T plot for compound 2 under the applied filed of 0.1 T (Figure S4).

S1. Powder X-ray diffraction (PXRD) of samples obtained from the reactions of compound 2.

Powder pattern of powder samples were collected from 5° to 50°, with a step of 0.02° using a Bruker D8 advance X–ray diffractometer with Cu–Kα radiation (λ = 1.54056 Å) equipped with a Lynxeye one-dimensional detector. Before PXRD measurement, sample for each reaction was fully grinded to avoid the strong diffraction from the preferential orientation in the crystal. Then, the powder sample was loaded on the glass tablet. The match between the experimental and simulated powder patterns indicates the examed sample of compound 2 is pure.

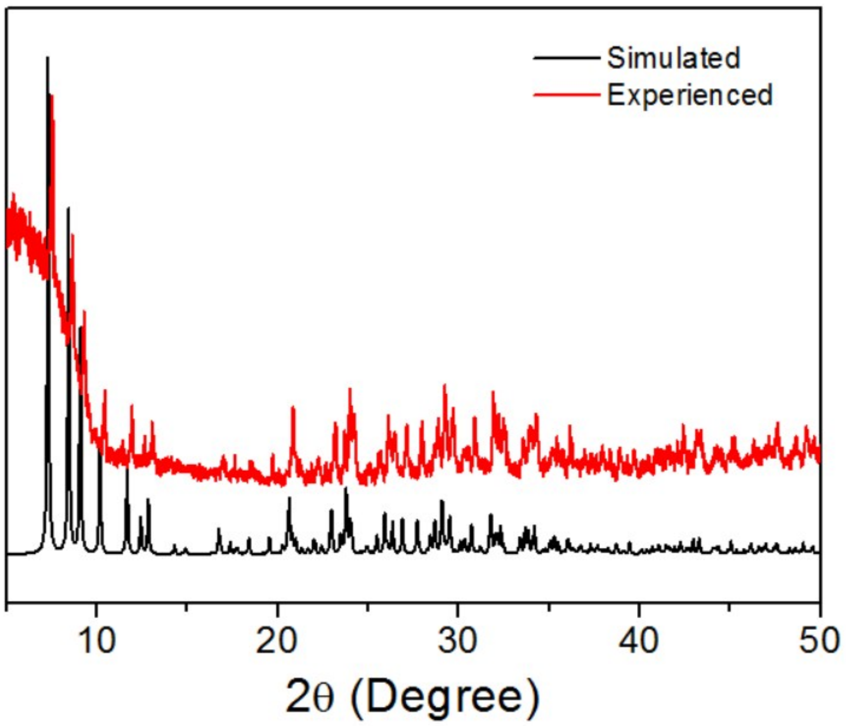

Figure S1. Simulated (black) and experimental (red) PXRD patterns for compound 2.

S2. Bond lengths between U(IV) and μ_3-O or μ_3-OH groups in three compounds (Table S1).

Compound 1a		Compound 1b		Compound 2	
U-O bond	bond length (Å)	U-O bond	bond length (Å)	U-O bond	bond length (Å)
O(6)-U(1)	2.222(6)	O(27)-U(1)	2.310(7)	O(1)-U(2)	2.219(4)
O(6)-U(2)	2.238(5)	O(27)-U(2)	2.243(8)	O(1)-U(3)	2.230(4)
O(6)-U(3)	2.240(5)	O(27)-U(5)	2.187(7)	O(1)-U(4)	2.288(4)
O(13)-U(2)	2.187(6)	O(31)-U(1)	2.275(7)	O(15)-U(1)	2.238(4)
O(13)-U(3)	2.244(6)	O(31)-U(3)	2.238(7)	O(15)-U(2)	2.255(4)
O(13)-U(4)	2.297(6)	O(31)-U(4)	2.204(7)	O(15)-U(3)	2.234(4)
O(3)-U(2)	2.419(6)	O(32)-U(2)	2.239(7)	O(7)-U(1)	2.432(4)
O(3)-U(3)	2.458(6)	O(32)-U(4)	2.233(7)	O(7)-U(2)	2.448(4)
O(3)-U(4)	2.427(6)	O(32)-U(6)	2.220(7)	O(7)-U(3)	2.464(4)
O(4)-U(1)	2.449(5)	O(33)-U(3)	2.222(7)	O(13)-U(2)	2.429(4)
O(4)-U(2)	2.444(6)	O(33)-U(5)	2.242(7)	O(13)-U(3)	2.390(4)
O(4)-U(3)	2.468(6)	O(33)-U(6)	2.227(7)	O(13)-U(4)	2.497(4)
		O(21)-U(1)	2.442(8)		
		O(21)-U(2)	2.452(7)		
		O(21)-U(4)	2.425(8)		
		O(22)-U(1)	2.424(8)		
		O(22)-U(3)	2.468(8)		
		O(22)-U(5)	2.408(7)		
		O(28)-U(3)	2.458(7)		
		O(28)-U(4)	2.455(8)		
		O(28)-U(6)	2.438(7)		
		O(30)-U(2)	2.479(7)		
		O(30)-U(5)	2.442(7)		
		O(30)-U(6)	2.460(7)		

S3. Bond valence sum (Σv, $v = e^{(R_0 - d)/a}$, d: bond distance) for μ_3-oxygen sites in compound 1a, 1b and 2 (R_0 = 2.051 Å and a = 0.51 for μ_3-O; R_0 = 2.104 Å and a = 0.37 for μ_3-OH).[1] (Table S2)

Compound 1a		Compound 1b		Compound 2	
μ_3-oxygen sites	Σv	μ_3-oxygen sites	Σv	μ_3-oxygen sites	Σv
O(6)	2.098	O(27)	2.064	O(1)	2.051
O(13)	2.068	O(31)	2.079	O(15)	2.061
O(3)H	1.229	O(32)	2.030	O(7)	1.185
O(4)H	1.187	O(33)	2.111	O(13)	1.223
		O(21)	1.211		
		O(22)	1.235		
		O(28)	1.176		
		O(30)	1.146		

[1] I. D. Brown, D. Altermatt, *Acta Crystallogr., Sect. B.*, 1985, **41**, 244-247.

S4. Magnetic susceptibility data of compound 2. (1) χ^{-1} - T plot for compound 2 under the applied filed of 0.1 T (Figure S3). (2) The inverse susceptibility data above 150 K was fitted to the C–W law (Figure S4). (3) χT-T plot for compound 2 under the applied filed of 0.1 T (Figure S5).

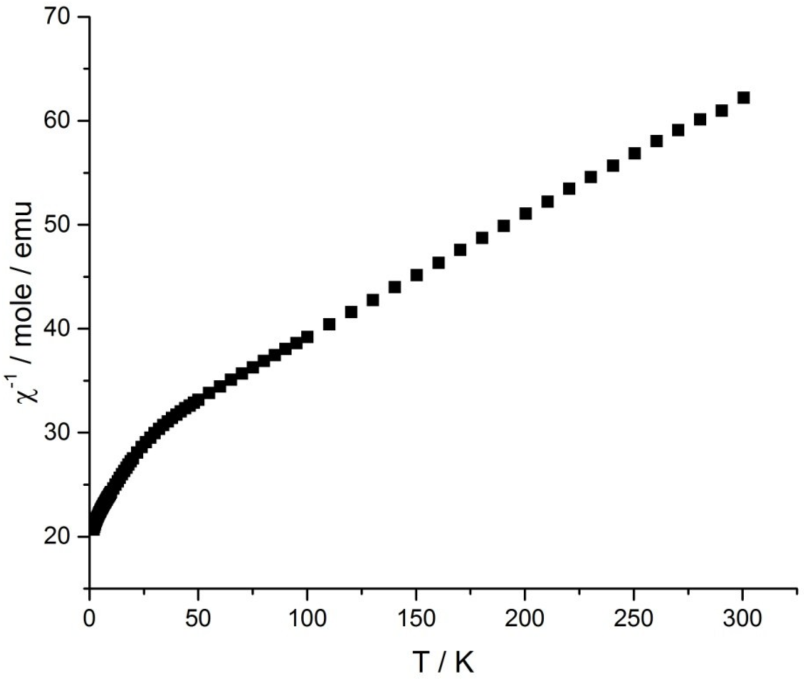

Figure S2. χ^{-1} - T plot for compound 2 under the applied filed of 0.1 T.

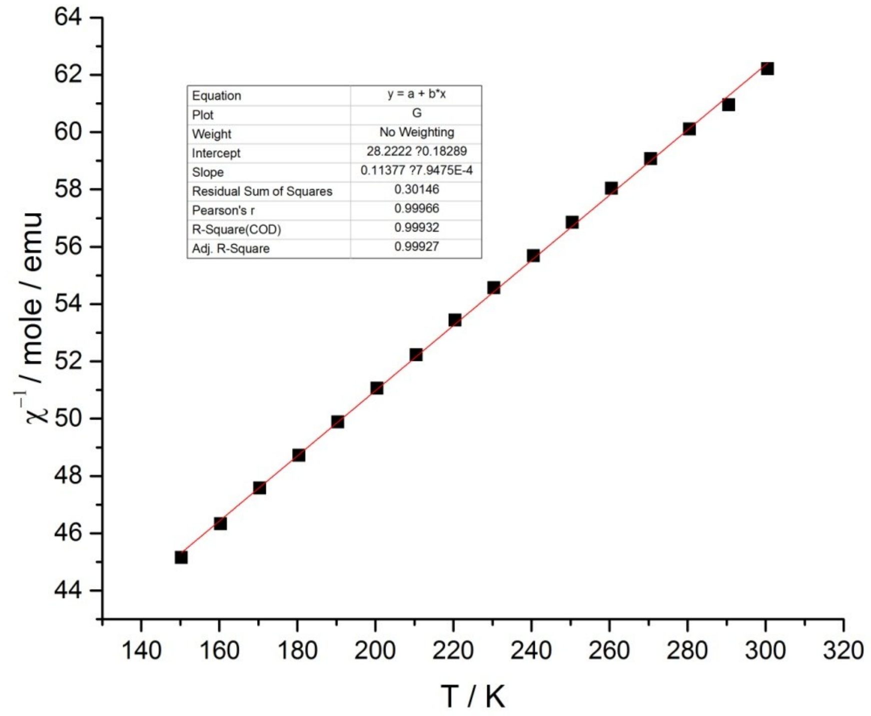

Figure S3. The inverse susceptibility data above 150 K was fitted to the C–W law (χ = C/(T - theta)), resulting in values of 8.790 emuK/mol and -248.07 K for the Curie and Weiss constants, respectively.

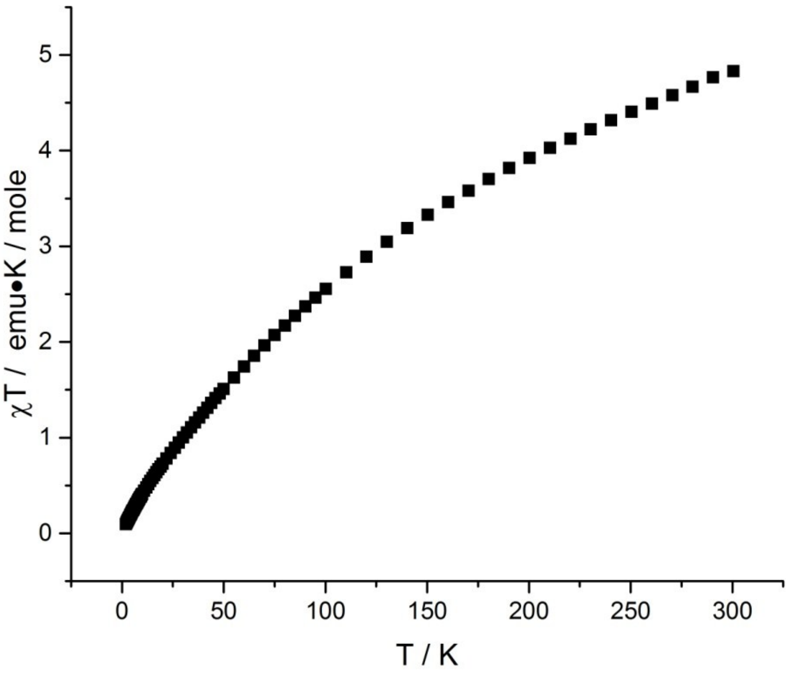

Figure S4. χT-T plot for compound 2 under the applied filed of 0.1 T.
